# Using ring‐recovery and within‐season recapture data to estimate fecundity and population growth

**DOI:** 10.1002/ece3.4506

**Published:** 2018-09-24

**Authors:** Todd W. Arnold

**Affiliations:** ^1^ Department of Fisheries, Wildlife, and Conservation Biology University of Minnesota St. Paul Minnesota

**Keywords:** Brownie band‐recovery model, recruitment, Seber tag‐recovery model

## Abstract

Tag‐recovery data from organisms captured and marked post breeding are commonly used to estimate juvenile and adult survival. If annual fecundity could also be estimated, tagging studies such as European and North American bird‐ringing schemes could provide all parameters needed to estimate population growth. I modified existing tag‐recovery models to allow estimation of annual fecundity using age composition and recapture probabilities obtained during routine banding operations of northern pintails (*Anas acuta*) and dark‐eyed juncos (*Junco hyemalis*), and I conducted simulations to assess estimator performance in relation to sample size. For pintails, population growth rate from band‐recovery data (λ = 0.93, *SD*: 0.06) was similar but less precise than count‐based estimates from the Waterfowl Breeding Pair and Habitat Survey (λ: 0.945, *SE*: 0.001). Models with temporal variation in vital rates indicated that annual population growth in pintails was driven primarily by variation in fecundity. Juncos had lower survival but greater fecundity, and their estimated population growth rate (λ: 1.01, *SD*: 0.19) was consistent with count‐based surveys (λ: 0.986). Simulations indicated that reliable (CV < 0.10) estimates of fecundity could be obtained with >1,000 within‐season live encounters. Although precision of survival estimates depended primarily on numbers of adult recoveries, estimates of fecundity and population growth were most sensitive to total number of live encounters. *Synthesis and applications:* Large‐scale ring‐recovery programs could be used to estimate annual fecundity in many species of birds, but the approach requires better data curation, including accurate assessment of age, better reporting of banding totals, and greater emphasis on obtaining and reporting within‐season live encounters.

## 1 INTRODUCTION

Tag‐recovery (a.k.a. ring‐ or band‐recovery) models are widely used to estimate annual survival using data on numbers of individuals surviving different intervals between tagging and reported time of death (Brownie, Anderson, Burnham, & Robson, 1978; Seber, 1970). Unlike live encounter data from restricted study areas, which provide estimates of apparent survival φ = (1 – mortality)*(1 – permanent emigration), dead recovery data can provide estimates of true survival *S *= (1 – mortality) provided that tag recoveries occur from throughout the population's potential dispersal or migratory range. This occurs most commonly with harvested populations of birds and fish (Brownie et al., 1978; Pollock, Hearn, & Polacheck, 2002), although dead‐recovery models have also been applied to unharvested species (Francis, 1995; Siriwardena, Baillie, & Wilson,^1998^). Dead recoveries can also be combined with live‐encounter data from restricted study areas to estimate true survival and permanent emigration (Barker, 1997; Burnham, 1993).

If individuals can be reliably assigned to age classes at the time of marking, tag‐recovery models can be used to estimate age‐specific survival (Brownie et al., 1978; Pollock et al., 2002; Seber, 1971). Most typically with birds, this approach has been used to provide age‐specific survival estimates for juveniles (*S*_j_) and adults (*S*_a_), but it can also be used for three or more age classes provided age classes can be recognized at marking (Brownie et al., 1978). For monogamous species that reach sexual maturity as one‐year‐olds and have limited sex‐ or age‐specific variation in survival or fecundity (e.g., many small birds and mammals), population dynamics can be modeled using a simple one‐stage projection model that captures most of the important variation in vital rates:

$$\begin{array}{c} N_{t+1}=N_{t}\left(S_{a,t}+F_{t}S_{j,t}\right) \end{array}$$

where *S*_a,t_ + *F*_t_ *S*_j,t_ is the population growth rate, $\lambda_{t}=N_{t+1}/N_{t}$ (Pulliam, 1988). Populations with additional nonbreeding stages could be readily modeled by extending this framework to include sub‐adult survival and life stages. Thus, tag‐recovery models can provide everything needed to estimate *λ*_t_ except annual fecundity *F*_t_.

Fecundity can be estimated using age ratios (*N*_j,t_/*N*_a,t_) collected during postbirth pulse surveys, and age ratios are commonly used when juveniles and adults can be readily distinguished during survey counts (Harris, Kauffman, & Mills, 2007; Weegman et al., 2016). Wildlife managers have long used age ratios of harvested individuals (*H*_j,t_/*H*_a,t_) to measure annual fecundity, but because juveniles are often more vulnerable to harvest than adults, tag‐recovery data are needed to adjust these data for relative vulnerability to harvest:

$${\hat{F}}_{t}=\frac{H_{j,t}/H_{a,t}}{{\hat{f}}_{j,t}/{\hat{f}}_{a,t}}$$

where ${\hat{f}}_{j,t}/{\hat{f}}_{a,t}$ is the ratio of juvenile to adult harvest rates (Zimmerman et al., 2010). Age ratios at capture can provide similar estimates of population‐level fecundity (Specht & Arnold, 2018), but if capture probabilities differ between age classes, fecundity estimates will be biased. However, live recaptures during the initial banding period could be used to assess age‐specific vulnerability to capture and estimate the true underlying age distribution, similarly to vulnerability‐adjusted age ratios at harvest (Alisauskas, Arnold, Leafloor, Otis, & Sedinger, 2014; Zimmerman et al., 2010). Even if estimation of capture vulnerability is not possible, age ratios at capture might nevertheless provide a reliable index of annual fecundity. Although uncorrected age ratios at capture have been used to assess population‐level fecundity (Mazerolle, Dufour, Hobson, & den Haan, 2005; Ross, Alisauskas, Douglas, & Kellett, 2017; Specht & Arnold, 2018), models to estimate fecundity from initial capture data have not been formally developed for tag‐recovery studies, although Link and Barker (2005) have addressed this issue for open‐population mark–recapture data.

**Figure 1:**
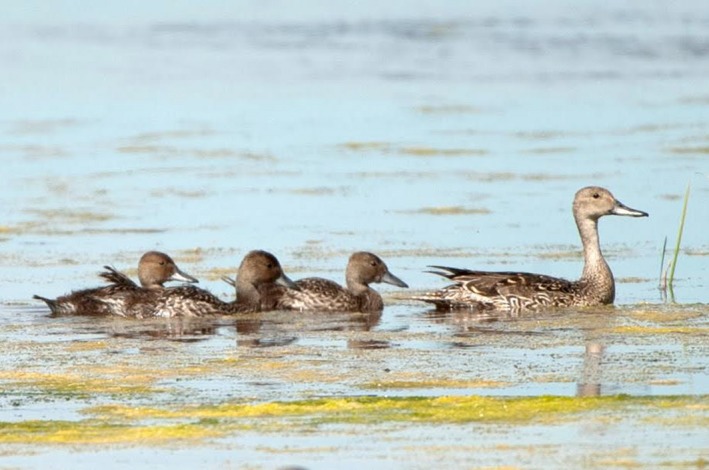
A female northern pintail (*Anas acuta*) with three nearly fledged ducklings. Photograph credit: Fred Greenslade, Delta Waterfowl

My objectives in this study are to develop and apply population projection models including fecundity, juvenile survival, and adult survival derived solely from tagging data (i.e., age‐specific counts of numbers of birds banded during postbreeding capture occasions, recaptured alive during the same season as originally marked or subsequently found dead any time after marking). I apply these models to two species of North American birds. Northern pintails (*Anas acuta*; Figure 1) have experienced a prolonged population decline and previous studies have shown it cannot be explained by declining survival (Bartzen & Dufour, 2017), suggesting that lowered fecundity may be the cause (Specht & Arnold, 2018), but to date there have been no integrated analyses for pintails to identify relative contributions of different vital rates to observed population changes (Koons, Arnold, & Schaub, 2017). Dark‐eyed juncos (*Junco hyemalis*) are a widespread passerine that has been well‐studied at several localized and primarily southern breeding sites (Nolan. et al., 2002), but most of their breeding range occurs in remote portions of the boreal forest that lie well north of established monitoring programs (Saracco, DeSante, & Kaschube, 2008; Sauer & Link, 2011). However, they are well sampled by migrant banding stations (Leppold & Mulvihill, 2011), so an approach that could estimate survival, fecundity, and population trajectory as birds pass southward during fall migration would be very useful for population monitoring, and could be applicable to numerous other Holarctic species with extreme northern breeding distributions (Hussell & Ralph, 2005; Spina, 1999). Model‐based fecundity estimates seemed reasonable for both pintails and juncos, but precision was poor given small numbers of within‐season recaptures, so I also conducted a simulation study to identify necessary sample sizes for obtaining more precise estimates. This approach provides new opportunities to estimate annual fecundity at local to continental scales and could greatly leverage the utility of existing banding data by allowing investigators to estimate a complete ensemble of vital rates from tagging studies.

## 2 MODEL AND METHODS

The model developed here assumes that animals are captured after the breeding season has ended using methods that are similarly effective at capturing adults and young of the year, and that captured individuals can be reliably aged at time of marking (e.g., Pyle, 1997).

### 2.1 Model development

A naïve estimator of annual fecundity that ignores differential vulnerability to capture is number of newly marked juvenile females divided by number of newly marked adult females *M*_jF_/*M*_aF_ (or for sexually monomorphic species, *M*_j_/*M*_a_). The equation for estimating age ratios from harvest data can be modified for tag‐recovery data as:

$${\hat{F}}_{t}=\frac{{\hat{N}}_{\mathrm{jF},t}}{{\hat{N}}_{\mathrm{aF},t}}=\frac{M_{\mathrm{jF},t}/M_{\mathrm{aF},t}}{{\hat{p}}_{\mathrm{jF},t}/{\hat{p}}_{\mathrm{aF},t}}$$

where ${\hat{p}}_{\mathrm{jF},t}$ is estimated capture probability for juvenile females in year *t*, ${\hat{p}}_{\mathrm{aF},t}$ is the equivalent parameter for adult females, and ${\hat{N}}_{\mathrm{jF},t}$ and ${\hat{N}}_{\mathrm{aF},t}$ are Horvitz and Thompson (1952) estimators of population size at time of capture. Any appropriate closed‐population mark–recapture model could be used to estimate capture probabilities, but given the sparseness of recapture data in my examples, I used Chao's (1989) estimator, which conditions on the number of individuals captured 1 versus 2 times. Under this model, vulnerability adjusted fecundity ($\hat{F}$) can be estimated as:

$$\hat{F}=\frac{{\hat{N}}_{\mathrm{jF}}}{{\hat{N}}_{\mathrm{aF}}}=\frac{M_{\mathrm{jF}}+{\left(f1_{\mathrm{jF}}\right)}^{2}/\left(2\times f2_{\mathrm{jF}}\right)}{M_{\mathrm{aF}}+{\left(f1_{\mathrm{aF}}\right)}^{2}/\left(2\times f2_{\mathrm{aF}}\right)}$$

where $\hat{F}$ is the estimated age ratio, ${\hat{N}}_{\mathrm{jF}}$ and ${\hat{N}}_{\mathrm{aF}}$ are estimated populations of juvenile and adult females that were available for capture, *M*_jF_ and *M*_aF_ were the total numbers of juveniles and adults captured and marked (i.e., *M*_jF_/*M*_aF_ is a naïve estimate of fecundity, estimated as $F_{\mathrm{naive}}=\frac{c_{\mathrm{jF}}}{1-c_{\mathrm{jF}}}$, where *c*_jF_ is the probability that an initial capture of a female will be juvenile: $M_{\mathrm{jF}}∼Bin\left(M_{\mathrm{jF}}+M_{\mathrm{aF}},c_{\mathrm{jF}}\right)$), and *f*1_jF_, *f*2_jF_, *f*1_aF_, and *f*2_aF_ were the numbers of juvenile and adult females captured 1 or 2 times, respectively (i.e., *M*_jF_ = *f*1_jF_ + *f*2_jF_, *f*2_jF_ ~ *Bin*(*M*_jF_, ${\hat{p}}_{\mathrm{jF}}$), *M*_aF_ = *f*1_aF_ + *f*2_aF_, *f*2_aF_ ~ *Bin*(*M*_aF_, ${\hat{p}}_{\mathrm{aF}}$)). Relative vulnerability ($\hat{V}$) to capture can then be estimated as:

$$\hat{V}=\frac{{\hat{p}}_{\mathrm{jF}}}{{\hat{p}}_{\mathrm{aF}}}=\frac{M_{\mathrm{jF}}/M_{\mathrm{aF}}}{\hat{F}}$$

Estimation of age ratios from live recapture data requires a similar set of assumptions as simple closed‐population mark‐recapture models (i.e. Model M_0_; Otis, Burnham, White, & Anderson, 1978), namely that: (a) the population is closed during sampling, (b) animals do not lose marks, (c) all marks are reported on discovery, (d) within age groups, all individuals have the same probability of capture, and (e) marking animals does not affect their subsequent catchability. These assumptions have been treated in detail elsewhere (Otis et al., 1978), so I focus here on potential violations specific to their application for estimating age ratios. To satisfy the closure assumption, analysts need to select appropriate marking periods for assessing postbirth pulse age structure; choosing intervals after young have become mobile, but before postbreeding dispersal or differential migration have altered local age ratios. If data are collected during migration, then ringing operations should include the entire migration period so that capture data are not affected by differential migration of adults versus juveniles (Kelly & Finch, 2000). Marker loss is negligible for within‐season recaptures, but ironically many North American banders do not report within‐season live encounters because the Bird Banding Laboratory historically discouraged such reports (Buckley et al., 1998). Homogeneity of capture probabilities among individuals and absence of behavioral response to capture are assumptions that can be accommodated under more elaborate models (Otis et al., 1978), but these assumptions are difficult to test with sparse data (Chao, 1989).

### 2.2 Application

**Figure 2:**
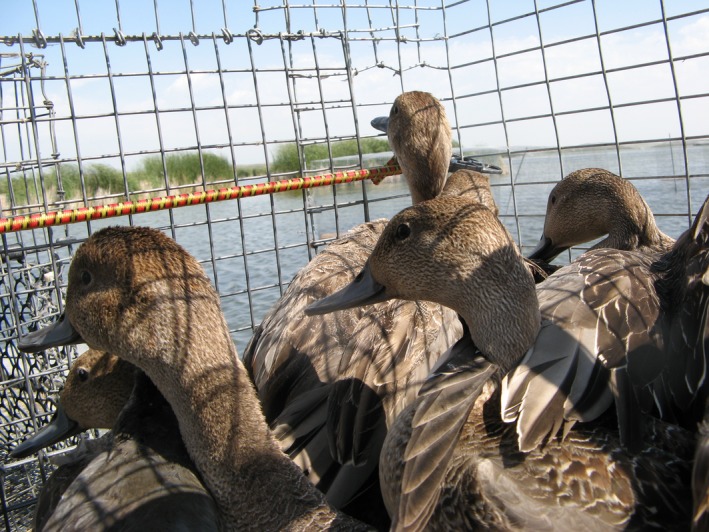
Age ratios of female northern pintails captured for banding could provide estimates of population‐level fecundity, but estimates should be corrected for potential differential vulnerability to capture. Photograph credit: David Johns

Examples used in this study include northern pintails captured primarily using bait traps (Figure 2) on their North American breeding grounds during July through September (Bartzen & Dufour, 2017) and dark‐eyed juncos captured primarily using mist nets throughout North America during August–October migration (Hussell & Ralph, 2005). I used data from 64,201 juvenile and 62,341 adult female northern pintails banded throughout the United States and Canada during 1970–1993 and shot or found dead during the hunting season (1 Sep‐31 Jan of year *t* + 1) 1970–1993 to assess performance of the fecundity model. In addition to the 3,841 and 2,377 dead recoveries obtained from juveniles and adults during subsequent hunting seasons, there were 90 and 44 live recaptures recorded during the initial banding season. For pintails, annual survey data were available from the Waterfowl Breeding Pair and Habitat Survey (U.S. Fish and Wildlife Service 2017), which indicated a severe population decline during this time period. For dark‐eyed juncos, data included 248,939 and 107,998 juveniles and adults banded between 1955 and 2013, but only 121 and 68 dead recoveries and 45 and 15 live encounters during the initial banding season.

I summarized data on number of bandings, dead recoveries, and live encounters during the initial trapping period into m‐arrays following standard procedures for band‐recovery analysis (Brownie et al., 1978), except I included an additional vector for recaptures (live encounters) during the initial capture period. For juncos, I summarized data in collapsed m‐array format recognizing only years since banding (Kéry & Schaub, 2012: 256) because data were too sparse to consider annual variation in survival or encounter probabilities. Summarized m‐arrays and additional details about the data, analysis, and JAGS code are provided as Supporting Information (Data S1).

As an initial template for analysis, I used Seber's (1971) model for estimating survival (*S*) and reporting rates (*r*) from dead‐recovery data, as coded by Kéry and Schaub (2012) for analysis in WinBUGS and further modified for analysis in JAGS 3.3.0 (Plummer, 2012) using the jagsUI package in R (Kellner, 2015). I first considered models that treated all parameters as constant through time (*S*_j_, *S*_a_, *r*_j_, *r*_a_, *p*_j_, *p*_a_, *c*_j_). For pintails, which had more extensive data, I also considered models that included temporal and age‐specific variation in survival, recovery, and initial capture probabilities (*S*_j,t_, *S*_a,t_, *r*_j,t_, *r*_a,t_, *c*_j,t_), but recapture data were too sparse so I treated capture probabilities (*p*_j_, *p*_a_) as age‐specific constants in all models. Time constant parameters were given vague uniform priors on the real scale (i.e., Uniform[0,1]), whereas temporally variable parameters were given vague priors on the logit scale (mean ~ Uniform[−2,2], *SD* ~ Uniform[0,2]). For pintails, I used an initial 1000 iteration adaptation phase, followed by three Markov chain Monte Carlo (MCMC) chains of 25,000 iterations each, with the first 5,000 iterations discarded as burn‐in, and retaining every 10th iteration for sampling from the posterior distribution. For juncos, I increased all iterations by 10‐fold to accommodate sparse data. Convergence was achieved for all parameters ($\hat{R}$ < 1.01) with run times of <1 min. Vulnerability (*V*), annual fecundity (*F*_t_) and finite population growth (*λ*_t_) were estimated as derived parameters:

$$V={\hat{p}}_{j}/{\hat{p}}_{a}$$

$$F_{t}=\frac{{\hat{c}}_{j,t}/\left(1-{\hat{c}}_{j,t}\right)}{V}$$

$$\lambda_{t}=S_{a,t}+F_{t}S_{j,t}$$

### 2.3 Simulation specifications

I conducted 1,000 24‐year simulations representing a data‐rich scenario patterned roughly on the northern pintail data. For each simulation I kept *S*_a_, *S*_j_ and *F* constant at 0.60, 0.50, and 0.80, respectively (hence, $\lambda =S_{a}+S_{j}F=1$), but varied number of recoveries and recaptures using random uniform distributions on *r* (*r*_j_ ~ U[0.0001, 0.4]; *r*_a_ ~ U[0.0001, 0.2]), *p* (*p*_a_ ~ U[0.0001, 0.02]) and *V* (U[0.5, 1.5], with *p*_j_ = *V* × *p*_a_) to produce varying numbers of bandings and live and dead encounters for fixed population sizes of *N*_j_ = 240,000 and *N*_a_ = 300,000. In addition to estimates of the mean and standard deviation (*SD*), I calculated bias, coefficient of variation (CV) and root mean‐squared error (RMSE = √[bias^2^ + *SD* ^2^]) for all population and encounter parameters. I compared the accuracy (RMSE) and precision (CV) of these estimates to numbers of juveniles and adults that were banded, recaptured during the first season following banding or recovered dead during their first or subsequent years to characterize how parameter estimates were affected by variation in quantity of data.

## 3 RESULTS

### 3.1 Case studies

**Figure 3:**
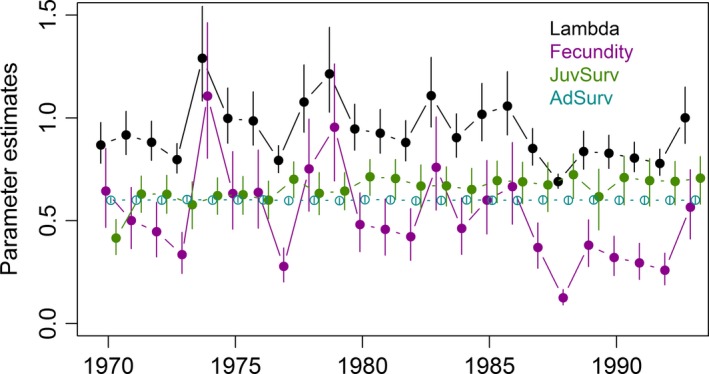
Annual estimates (90% credible intervals) of juvenile survival (JuvSurv; green, closed symbols), adult survival (AdSurv; blue‐green, open symbols), fecundity (purple) and annual population growth (Lambda; black) for northern pintails during 1970–1993. Fecundity was estimated from age ratios at capture and explained most of the annual variation in lambda

Juvenile pintails were more likely to be recaptured than adults ($\hat{V}$ = 2.05, 90% credible interval [CRI]: 1.49–2.73), but uncertainty in this parameter translated into large uncertainty in estimates of adjusted fecundity and population growth rate. In the simplest model with no temporal variation in survival and recovery rates, unadjusted age ratios and *λ* were precisely estimated (CV < 0.1); but recapture rates, vulnerability, and adjusted age ratios all had CVs between 0.1 and 0.2 (Table 1). In a fully temporal model, adult survival averaged 0.601 with essentially no annual variation (*SD*_t_ = 0.002), juvenile survival averaged 0.654 with modest annual variation (*SD*_t_ = 0.064) and fecundity averaged 0.520 with extensive annual variation (*SD*_t_ = 0.227). Annual variation in λ_*t*_ was strongly correlated with estimated fecundity (Pearson's *r *=* *0.97), but not with adult or juvenile survival (Figure 3). Mean annual population growth under both models (time constant: λ = 0.93, 90% CRI: 0.84–1.04; time varying: *t* = 0.94, *SD*_t_ = 0.14) included the estimate derived from survey data ($\hat{\lambda }$ = 0.945, *SE* = 0.001).

**Table 1:** Estimates of juvenile (juv) and adult (ad) annual survival (*S*), dead recovery (*r*) and live recapture (*p*) probabilities and associated estimates of capture vulnerability (*V*), age ratios at capture (*M*_j_/*M*_a_), fecundity (*F*) and finite population growth (*λ*) for northern pintails and dark‐eyed juncos under time‐constant models

	Northern pintails			Dark‐eyed juncos		
	Mean	*SD*	CV	Mean	*SD*	CV
*S*.juv	0.629	0.021	0.033	0.276	0.055	0.200
*S*.ad	0.613	0.0054	0.009	0.493	0.034	0.070
*r*.juv	0.097	0.006	0.061	0.00045	0.00006	0.138
*r*.ad	0.0412	0.0008	0.020	0.00063	0.00008	0.122
*p*.juv	0.00144	0.00015	0.105	0.00019	0.00003	0.147
*p*.ad	0.00072	0.00011	0.153	0.00015	0.00004	0.250
*V*	2.04	0.38	0.187	1.330	0.410	0.308
*M*_j_/*M*_a_	0.992	0.0057	0.006	2.305	0.008	0.004
*F*	0.503	0.094	0.186	1.889	0.556	0.294
λ	0.929	0.060	0.064	1.014	0.191	0.189

For juncos, juvenile vulnerability to capture was imprecisely estimated with a credible interval that overlapped 1 ($\hat{V}$ = 1.33, 90% CRI: 0.79–2.10). Only unadjusted (raw) age ratios and adult survival were precisely estimated (CV < 0.1), with remaining parameters having CVs exceeding 0.12 (Table 1). Estimated λ was 1.015 (90% CRI: 0.755–1.371), which included the continental estimates based on Breeding Bird Survey data ($\hat{\lambda }$ 0.989, 95% CRI: 0.983–0.995; Sauer & Link, 2011) and constant‐effort ringing stations ($\hat{\lambda }$ 1.007, 95% CI: 0.997–1.017; DeSante, Kaschube, & Saracco, 2015).

### 3.2 Simulations

**Figure 4:**
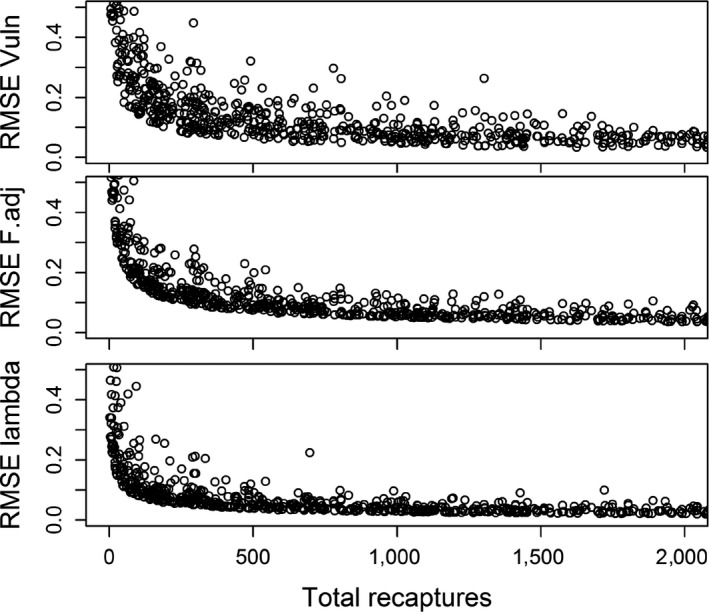
Effect of number of live recaptures (combined juvenile and adult) during the initial marking period on root mean‐squared error (RMSE) of vulnerability to capture (Vuln), annual fecundity (*F*.adj) and population growth rate (lambda). Note that age composition of recaptures (0.5–1.5 juveniles per adult) and dead recovery probability (0–0.4 for juveniles, 0–0.2 for adults) also varied randomly among simulations, adding to among‐replicate variation

Precision and accuracy (i.e., lower CV and RMSE, respectively) of juvenile and adult survival and recovery probabilities increased with increasing juvenile, adult, and total recoveries, but these relationships were strongest for adult recoveries (Figure S1). Accuracy of vulnerability and adjusted fecundity was most strongly affected by total number of live encounters (Figure 4). Reasonable estimates (CV < 0.20) of these two parameters required >300 total recaptures, whereas precise estimates (CV < 0.10) required >1,000 total recaptures. Because estimates of population growth (*λ*) depended on both survival and fecundity, accuracy of λ estimates were affected by both recoveries and recaptures, but recaptures had a stronger influence.

## 4 DISCUSSION

Using empirical data on age ratios at capture for northern pintails and dark‐eyed juncos, I was able to obtain estimates of annual fecundity by adjusting for vulnerability to capture using live encounters obtained during the original banding season. These fecundity estimates complemented estimates of juvenile and adult survival that analysts have typically obtained from tag‐recovery data (Brownie et al., 1978; Siriwardena et al., 1998) and allowed me to construct models that included all of the demographic components of population growth. For pintails, data were sufficient to estimate annual variation in all three vital rates and these estimates suggested that observed population declines during 1970–1993 were driven primarily by reductions in annual fecundity, which is consistent with other recent studies of historical pintail data (Bartzen & Dufour, 2017; Specht & Arnold, 2018). For juncos, my estimate of adult survival (0.493, *SD*: 0.034) was slightly larger than estimates of apparent survival from constant‐effort ringing stations (0.453, *SD*: 0.030; DeSante et al., 2015), whereas my estimate of recruitment (*F* × *S*_juv_ = 1.889 × 0.276 = 0.521) was slightly lower than estimates derived from reverse‐time analyses (0.554, *SD*: 0.032; DeSante et al., 2015). Both of these slight differences are consistent with the realization that in Pradel (1996) models, apparent survival estimates include permanent emigration and mortality, whereas recruitment estimates include both immigration and fecundity.

Avian ecologists often have access to large‐scale count data to assess annual fluctuations in population size (Newson, Evans, Noble, Greenwood, & Gaston, 2008; Sauer & Link, 2011) and continental banding or ringing programs can provide similar data on age‐specific survival or apparent survival (Francis, 1995; Saracco, Royle, DeSante, & Gardner, 2010; Siriwardena et al., 1998), but fecundity data are often lacking (Ahrestani, Saracco, Sauer, Pardieck, & Royle, 2017). To assess fecundity, population modelers have used age ratios at harvest from hunted species (Péron & Koons, 2012), fledgling counts from citizen‐scientist nest‐record programs (Robinson, Morrison, & Baillie, 2014), data from small‐scale nesting studies (Weegman, Arnold, Dawson, Winkler, & Clark, 2017) and reverse‐time mark–recapture models (which measure the product of fecundity and first‐year survival; Pradel, 1996; Saracco et al., 2008), but age ratios at capture could provide an alternative or complementary data stream to assess spatiotemporal variation in fecundity (Mazerolle et al., 2005; Ross et al., 2017; Specht & Arnold, 2018). In the absence of live recapture data, vulnerability to capture (*V*) could be estimated in an integrated population modeling (IPM) framework (Ahrestani et al., 2017), assuming that auxiliary population count data were available and that there were no confounding influences of immigration or emigration:

$$N_{t+1}=N_{t}{\left[S_{a}+\left(S_{j}M_{j}\right)/\left({\text{VM}}_{a}\right)\right]}_{t}$$

If marking efforts occur at the end of the breeding season, but before postbreeding dispersal or migration, then age ratios at marking measure local reproductive success and spatially extensive marking data have the potential to measure regional variation in fecundity and identify ecological or anthropogenic drivers of this variation (Specht & Arnold, 2018). However, researchers must have a thorough understanding of breeding and movement phenology to select appropriate intervals and spatial scales for data analysis, thereby assuring that age ratios are not affected by ongoing breeding efforts or early dispersal or migration by one age class versus another (Andres, Browne, & Brann, 2005). Variation in age ratios at capture could also be due to age‐related variation in local habitat use on the breeding grounds, especially if capture efforts are not randomly distributed among potential habitats. Treating individual capture sites as random effects could potentially control for some of this location‐specific variation (Specht & Arnold, 2018) and testing for seasonal trends in age ratios could help identify ongoing breeding or differential movements. Age ratios might also be affected by capture methods, if juveniles are more (or less) vulnerable to capture by widely employed capture methods. In North America, relatively few capture methods are uniquely coded at time of banding (https://www.pwrc.usgs.gov/BBL/MANUAL/summary.cfm), but European ringing schemes record a wide diversity of capture methods and lure types (https://euring.org/files/documents/E2000PLUSExchangeCodev1161.pdf), thereby allowing for a thorough investigation of heterogeneity in age ratios induced by capture methodology.

In the northern hemisphere, many birds are banded or ringed during autumn as they migrate from northern hemisphere breeding sites to equatorial or southern hemisphere wintering sites (Hussell & Ralph, 2005; Spina, 1999). Such marking programs have the potential to assess continental‐level productivity, but meeting the closure assumption seems much more difficult in this situation (Hochachka & Fiedler, 2008). Nichols, Thomas, and Conn (2009) partitioned detection probability from count surveys into four conditional components, and a similar hierarchy could be extended to same‐season capture probabilities. First, choice of marking sites could affect age ratios at capture if juveniles and adults have different migration routes (Ralph, 1978). Second, differential timing of migration could affect age ratios at capture (Andres et al., 2005), especially if one age class exhibits a more prolonged migration and capture efforts are limited to periods of peak migration. Third, age‐related capture probability could be affected by differences in stopover durations; for example, if juveniles spend more time “refueling” at migrational stopover sites they would be more vulnerable to capture (Rguibi‐Idrissi, Julliard, & Bairlein, 2003), especially if permanent marking sites are concentrated at migrational stopover sites. Finally, because juveniles are more naïve, they may be more vulnerable to capture by standard trapping methods (Rguibi‐Idrissi et al., 2003), even if locations and timing were otherwise unbiased.

Probably the biggest limitation to employing the fecundity estimation approach developed herein is the paucity of within‐season live‐encounter data for estimating vulnerability to initial capture. With sufficient recapture data, many of these assumptions could be tested, and some ringing stations have sufficient in‐house data to estimate capture vulnerability (e.g., Hochachka & Fiedler, 2008). During routine duck banding operations in Alberta, Canada, approximately 54% of 33,552 ducks captured for banding over a three‐year period were within‐season recaptures (Dieter, Murano, & Galster, 2009), but banding crews have not been encouraged to collect and report these data. North American banders were historically dissuaded from reporting same‐station live encounters, and hence, live encounter data are limiting for historical analyses, although this shortcoming has been recently corrected (Smith, 2013) and many North American banders have begun submitting large amounts of recapture data (D. Bystrak, Patuxent Wildlife Research Center, pers. comm.). In Europe, many national ringing programs failed to keep records of numbers of bands deployed and focused primarily on banding known‐age juveniles, but this shortcoming was recognized in the mid‐1980s (e.g., Anderson, Burnham, & White, 1985) and ringing schemes have since expanded to include adults, and historical summaries of ring deployment have since been compiled for many European countries going back to 1975 (https://euring.org/data-and-codes/ringing-totals). Bird ringers need to be made aware of the value of live encounters, even those from the same location and banding season, and national banding programs need to be made aware of the value of collecting and archiving such data. The ability to estimate fecundity from age ratios at the time of marking greatly enhances the utility of continental ringing programs, because it allows important vital rates to be estimated as markers are deployed, while investigators wait for encounter data to accumulate.

## AUTHOR CONTRIBUTIONS

TWA conceived the idea, collated and analyzed data, developed code, and wrote the manuscript.

## DATA ACCESSIBLITY

Data and code used for analysis and simulations are included as Supporting Information (Data S1).
