# Supplementary material for: Using ring‐recovery and within‐season recapture data to estimate fecundity and population growth
Source: Ecol Evol. 2018 Sep 24;8(20):10298–305. doi: 10.1002/ece3.4506 (PMC6206198; doi:10.1002/ece3.4506)
Supplement: Supplementary file 1 [file ECE3-8-10298-s001.docx]

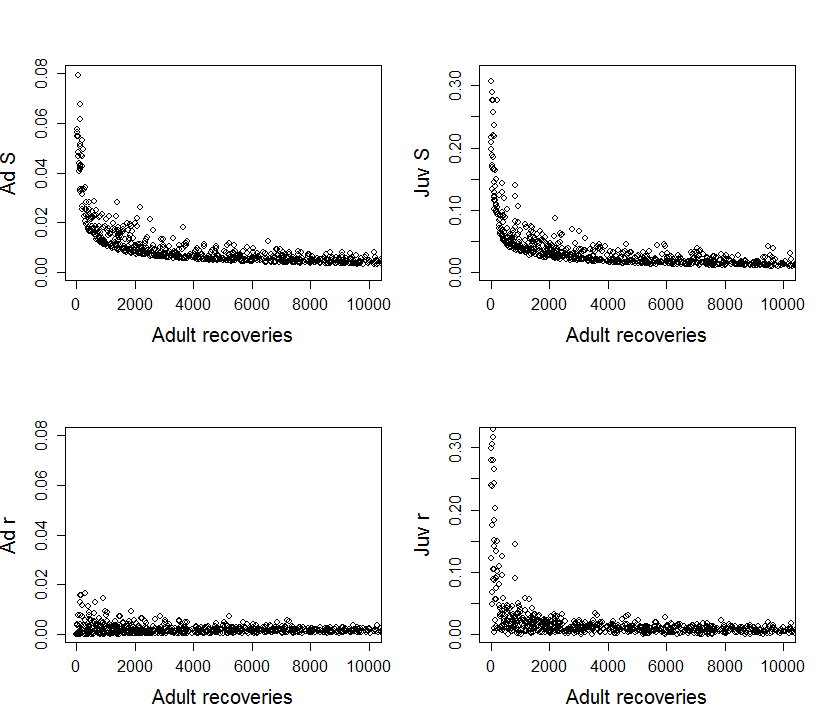

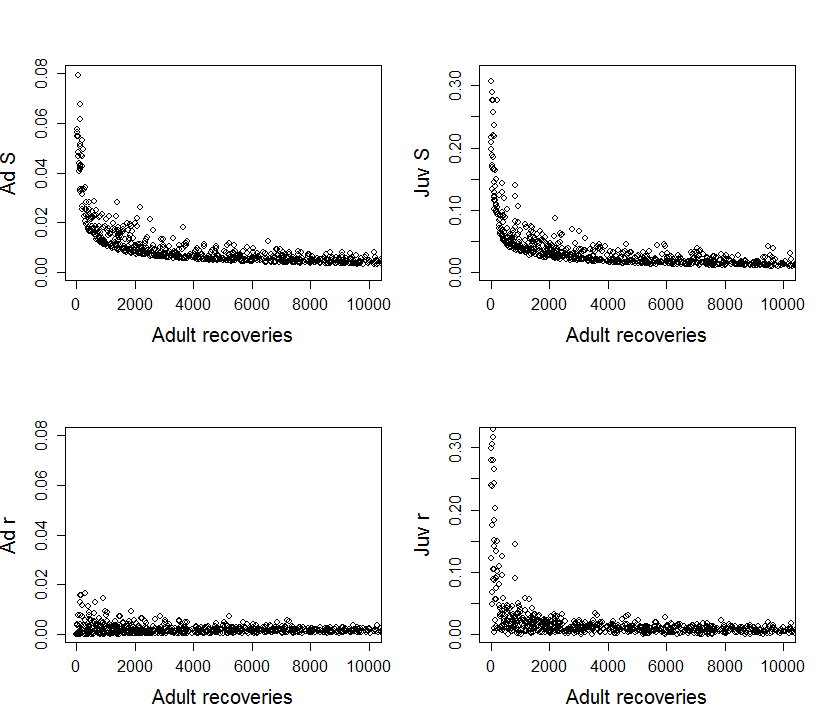


Fig S1: Effect of number of adult recoveries on accuracy (root mean-squared error) of juvenile and adult survival and reporting rates from Seber recovery models. Note the RMSE scale for juveniles (right column) is 4-fold higher than for adults. In addition to variation in adult recoveries, juvenile recovery probabilities also varied randomly from 0 to 0.4 across all simulations, contributing additional variation.
